# Re-sequencing of the complete chloroplast genome of *Cinnamomum burmanni *(Nees & T.Nees) Blume (Lauraceae) from Indonesia using MinION Oxford Nanopore Technologies

**DOI:** 10.12688/f1000research.145790.1

**Published:** 2024-02-20

**Authors:** Richard Andreas Salindeho, Fifi Gus Dwiyanti, Rahadian Pratama, Deden Derajat Matra, Muhammad Majiidu, Iskandar Z. Siregar

**Affiliations:** 1 Department of Silviculture, Faculty of Forestry and Environment, IPB University, Bogor, West Java, 16680, Indonesia; 2 Molecular Science Research Group, Advanced Research Laboratory, IPB University, Bogor, West Java, 16680, Indonesia; 3 Department of Biochemistry, Faculty of Mathematics and Natural Sciences, IPB University, Bogor, West Java, 16680, Indonesia; 4 Department of Agronomy and Horticulture, Faculty of Agriculture, IPB University, Bogor, West Java, 16680, Indonesia

**Keywords:** Conservation, complete chloroplast genome, Lauraceae, phylogenetic

## Abstract

*Cinnamomum burmanni* (Nees & T.Nees) Blume (Lauraceae) belongs to the Magnoliids group and is mainly distributed in Indonesia and Southeast Asia. The complete chloroplast (cp) genome of *C. burmanni* sampled from Indonesia was assembled and annotated for the first time in this study. The length of the cp genome is 152,765 bp with a GC content of 39%, and it consists of four subregions: a large single-copy (LSC) region of 93,636 bp, a small single-copy (SSC) region of 18,893 bp and two inverted repeats (IR) regions (IRA 20,121 bp; IRB 20,115 bp) . The cp genome of *C. burmanni* encodes a total of 173 unique genes, which are 96 protein-coding genes, 19 rRNA genes, and 68 tRNA genes that can be utilized for advanced genetic and genomic studies of the species.

## Introduction

*Cinnamomum burmanni* (or cinnamon) is an endemic woody shrub belonging to the Lauraceae family, which is widely distributed in Indonesia covering West Sumatra, North Sumatra, Jambi, Bengkulu, Java Island, and Maluku Islands ( Suwarto *et al.*, 2014). The species grows in altitudes between 0 and 2000 m above sea level. The tree can grow up to 15 m tall, and the wood is grey with a very distinctive aroma and sweet taste, so its wood is widely used for spices, cosmetics, and herbs. The active compounds contained in cinnamon wood are cinnamaldehyde, flavonoids, alkaloids, tannins, saponins, coumarins, steroids, eugenol, and phenols, which act as anti-bacterial, anti-tumor, antioxidant, anti-inflammatory, anti-cancer, and anti-diabetic agents ( Indarto *et al.*, 2022).

The chloroplast (cp) genome sequence of *C. burmanni* from China has been previously generated by Yang *et al.* (2019) using 11 universal primer pairs to perform long-range PCR for next-generation sequencing. However, the study of the cp genome *C. burmanni* from Indonesia using PCR-free library preparation method and long-read sequencing generated by MinION Oxford Nanopore Technologies (ONT) has not been carried out, whereas this information is needed to improve correct species identification and to optimize the sustainable use of genetic resources for this species. MinION is a third-generation sequencing technology with nanopore technology ( Oxford Nanopore Technologies (ONT), 2017). Furthermore, the present study aimed to re-sequence and assemble the complete cp genome *C. burmanni* from Indonesia using MinION Oxford Nanopore Technologies (ONT).

## Methods

Fresh leaf samples were collected from one individual *C. burmanni* tree in Lembang Subdistrict, West Bandung Regency, West Java Province, Indonesia. The collected leaf sample was subsequently used for DNA extraction and sequencing in the field. The data analysis of the chloroplast genome was performed at the Laboratory of Forest Genetics and Molecular Forestry, Department of Silviculture, Faculty of Forestry and Environment, IPB University.

*C. burmanni* genomic DNA was extracted using the Qiagen DNeasy Plant Mini Kit (cat. nos. 69104 and 69106) following the protocol provided by the manufacturer with slight modifications. Extraction was carried out first by grinding fresh leaf samples to which 400 μl of Buffer AP1 and 1 μl of mercaptoethanol had been added using a mortar and pestle. The disrupted sample was placed into a 1.5 ml microcentrifuge tube. The mixture was then incubated for 10 min at 65°C using Mini Heating Dry Bath Incubator MD-MINI (Major Science Co., Ltd). Afterward, 130 μl Buffer P3 was added into the microtube and then vortexed using Biosan V-32 Multi-Vortex and incubated for 5 min on ice. The mixture was centrifuged for 2 min at 8000 × g using a portable microcentrifuge on the Bento Lab device (Bento Bioworks Ltd). The lysate was pipetted into a QIAshredder spin column placed in a 2 ml collection tube. The lysate was centrifuged for 2 min at 8000 × g using a portable microcentrifuge on the Bento Lab device (Bento Bioworks Ltd). The flow-through was transferred into a new tube without disturbing the DNA pellet. 1.5 volumes of Buffer AW1 were then added and mixed by pipetting. 650 μl of the mixture was transferred into a DNeasy Mini spin column placed in a 2 ml collection tube and subsequently centrifuged for 1 min at 6000 × g. The flow-through was discarded and the spin column was placed into a new 2 ml collection tube. 500 μl Buffer AW2 was added to the spin column and centrifuged for 1 min at 6000 × g. The flow-through was then discarded and the spin column was transferred to a new 1.5 ml microcentrifuge tube. 50 μl Buffer AE was added for elution and subsequently incubated for 5 min at room temperature (15–25°C) before centrifuging for 1 min at 6000 × g. The quantity of genomic DNA was measured by the Invitrogen Qubit 1.0 fluorometer with the Qubit dsDNA BR assay kit.

The high molecular weight of 400 ng evaluated DNA proceeded to PCR-free library preparation, which followed the Nanopore protocol for Rapid sequencing gDNA – Field Sequencing Kit (SQK-LRK 001) version FSK_9049_v1_revR_14Aug2019. The prepared DNA library was sequenced using the MinION R9.4.1 flow cell (FLO-MIN106D) on a MinION Mk1B sequencer (Oxford Nanopore Technologies). The runs were visualized by MinKnow v3.6.5 software ( http://community.nanoporetech.com Oxford Nanopore Technologies).

Basecalling with super accuracy mode (SUP) to translate Fast5 raw data into Fastq (DNA-Seq of *Cinnamomum burmanni.* NCBI Accession number SRX22198906) data was performed using the Guppy program (RRID:SCR_023196) v4.2.3+8aca2af8 ( Wick *et al.*, 2019). Quality control was done using NanoStat v1.5.0 and NanoPlot (RRID:SCR_024128) v1.28.2 ( De Coster *et al.*, 2018). Chloroplast genome assembly was performed using Galaxy Server (RRID:SCR_006281) version 23.1.1.dev0 ( Sloggett *et al.*, 2013). The assembly includes a filtering step to filter reads quality (Q>7) and length minimum of reads (>500 bp), using the Flye program (RRID:SCR_017016) v2.9 ( Kolmogorov *et al.*, 2020) and polishing the assembly results using the MEDAKA consensus program (RRID:SCR_005857) v1.4.4 ( Oosterbroek *et al.*, 2021). The cpDNA data was then annotated using the GeSeq (RRID:SCR_017336) on the CHLOROBOX platform ( Tillich *et al.*, 2017) to assign functions to the predicted genes and generate a map representation of the chloroplast genome.

## Results

**Figure 1.**
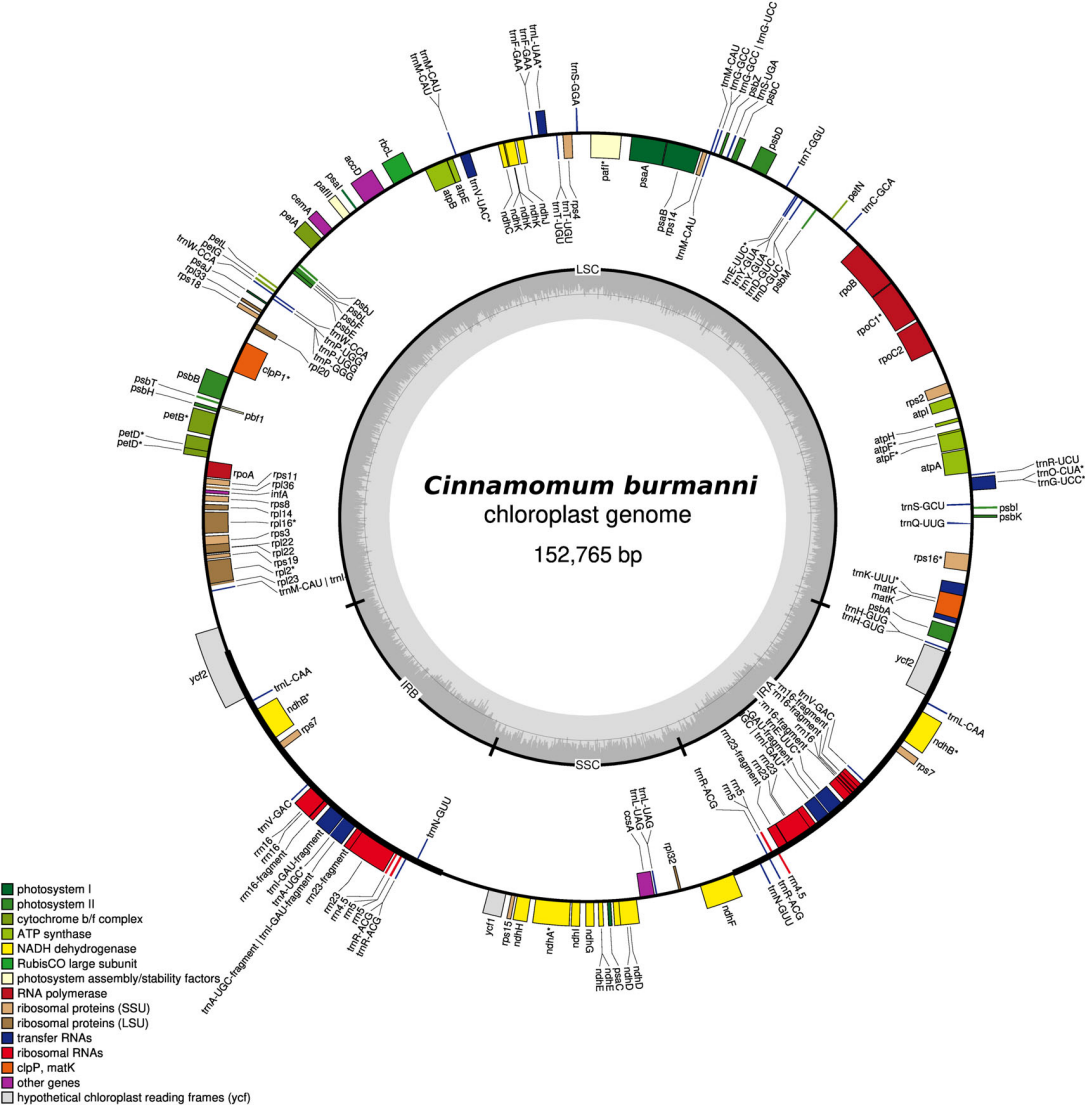
The complete chloroplast genome of *Cinnamomum burmanni.*

The cp genome showed a typical quadripartite structure ( Figure 1) with a length of 152,765 bp, consisting of small single copy (SSC 18,893 bp) and large single copy (LSC 93,636 bp) regions separated by a pair of inverted repeat A (IRA 20,121 bp) and inverted repeat B (IRB 20,115 bp) regions ( Figure 1). The *C. burmanni* chloroplast genome contained 173 unique genes, including 96 coding sequences, 68 transfer RNA (tRNA), and 19 ribosomal RNA (rRNA) genes ( Table 1). The *C. burmanni* sequence had a GC content of 39% (LSC 38%; SSC 34%; IR 44%). The results of the typical quadripartite structure were similar to the *C. burmanni* reported by Yang *et al.* (2019).

**Table 1.** List of genes in the chloroplast genome of *Cinnamomum burmanni.*

Functional category	Group of genes	Name of genes
Self-replication	Large subunit ribosomal proteins	*rpl32, rpl33, rpl20, rpl36, rpl14, rpl16* *(×2) *, rpl22, rpl2* *(×2) *, rpl23*
	DNA dependent RNA polymerase	*rpoC2, rpoC1* *(×2) *, rpoB, rpoA*
	Small subunit ribosomal proteins	*rps7, rps12* **(×2) *, rps15, rps7, rps16* *(×2) *, rps2, rps14, rps4, rps18, rps11, rps8, rps3, rps19, rps12-*fragment *
	rRNAs	*rrn16, rrn23, rrn5, rrn4.5*
	tRNAs	*trnL-CAA, trnV-GAC, trnE-UUC, trnA-UGC* *(×2) *, trnN-GUU, trnL-UAG, trnN-GUU, trnR-ACG, trnR-ACG, trnI-GAU* *(×2) *, trnV-GAC, trnL-CAA, trnH-GUG, trnK-UUU* *(×2) *, trnQ-UUG, trnG-UCC* *(×2) *, trnR-UCU, trnC-GCA, trnD-GUC, trnY-GUA, trnT-GGU, trnS-UGA, trnG-GCC, trnM-CAU, trnfM-CAU, trnS-GGA, trnT-UGU, trnL-UAA* *(×2) *, trnF-GAA, trnV-UAC* *(×2) *, trnW-CCA, trnP-UGG, trnI-CAU, trnS-GCU*
	Subunit of ATP synthase	*atpA, atpF* *(×2) *, atpH, atpI, atpE, atpB*
	Subunit of NADH-dehydrogenase	*ndhB* *(×2) *, ndhH, ndhA* *(×2) *, ndhI, ndhG, ndhE, ndhD, ndhF, ndhJ, ndhK, ndhC*
Photosynthesis	Subunits of cytochrome b/f complex	*petN, petA, petL, petG, petB* *(×2) *, petD* *(×2)
	Subunits of photosystem I	*psaC, psaB, psaA, psaI, psaJ*
	Subunits of photosystem II	*psbK, psbI, psbM, psbD, psbC, psbZ, psbJ, psbL, psbF, psbE, psbB, psbT, psbH, psbA*
	Subunit rubisco	*rbcL*
	Photosystem assembly factors	*pafI* **(×2) *, pafII*
	Photosystem biogenesis factor	*Pbf1*
	Subunit of acetyl-CoA-carboxylase	*accD*
	C-type cytochrome synthesis gene	*ccsA*
Other functions	Envelope membrane protein	*cemA*
	ATP-dependent protease subunit P	*clpP1* **(×2)
	Maturase	*matK*
	Conserved open reading frames	*ycf2* (×2)
	Transitional initiation factor	*infA*

* Gene containing single intron.

** Gene containing two introns.

Genes located in the IR regions are indicated by the (×2) symbol.

## Data Availability

NCBI’s Short Read Archive (SRA): DNA-Seq of *Cinnamomum burmanni.* Accession number SRX22198906; https://identifiers.org/insdc.sra:SRX22198906 ( IPB University, 2023).
