# Supplementary material for: Re-sequencing of the complete chloroplast genome of Cinnamomum burmanni (Nees & T.Nees) Blume (Lauraceae) from Indonesia using MinION Oxford Nanopore Technologies
Source: F1000Res. 2025 Dec 16;13:127. Originally published 2024 Feb 20. [Version 3] doi: 10.12688/f1000research.145790.3 (PMC12790596; doi:10.12688/f1000research.145790.3)
Supplement: Peer review history

# Reviewer report: Reviewer response for version 3

**DOI:** 10.5256/f1000research.193459.r441776

**Published:** 2026-01-12

**Authors:** Minh Trong Quang

**Affiliations:** 1 University of Medicine and Pharmacy, Ho Chi Minh City, Vietnam

I have carefully reviewed the revised version of the manuscript as well as the authors’ responses to previous reviewer comments. The authors have addressed the major technical and presentation-related concerns raised during earlier review rounds, including improvements to the Introduction, clarification of the sequencing and assembly workflow, correction of inconsistencies between Figure 1 and Table 1, and clearer reporting of chloroplast genome structure and gene content. The methodological description is now more focused, and the Results section more clearly reports key assembly statistics and genome features.

 While some limitations inherent to a Genome Note remain (e.g., limited comparative and evolutionary interpretation), these are now appropriately aligned with the scope and aims of the article.

 Overall, the revised manuscript represents a technically sound and useful genomic resource that adds geographic representation for *Cinnamomum burmanni* plastomes and should be of value to researchers working in plant genomics and chloroplast genome studies.

 I therefore have **no further major comments** to make on this revised version.

Are the datasets clearly presented in a usable and accessible format, and the assembly and annotation available in an appropriate subject-specific repository?

Partly

Are sufficient details of the sequencing and extraction, software used, and materials provided to allow replication by others?

Partly

Are the rationale for sequencing the genome and the species significance clearly described?

Partly

Are the protocols appropriate and is the work technically sound?

Partly

Reviewer Expertise:

Bioinformatics, Genetics, Biotechnology

I confirm that I have read this submission and believe that I have an appropriate level of expertise to confirm that it is of an acceptable scientific standard.

# Reviewer report: Reviewer response for version 3

**DOI:** 10.5256/f1000research.193459.r441775

**Published:** 2026-01-10

**Authors:** Jakub Sawicki

**Affiliations:** 1 Department of Botany and Nature Protection, University of Warmia and Mazury in Olsztyn, Olsztyn, Poland

The authors resolved all raised issues. I recommend acceptance.

Are the datasets clearly presented in a usable and accessible format, and the assembly and annotation available in an appropriate subject-specific repository?

Partly

Are sufficient details of the sequencing and extraction, software used, and materials provided to allow replication by others?

Partly

Are the rationale for sequencing the genome and the species significance clearly described?

Partly

Are the protocols appropriate and is the work technically sound?

Partly

Reviewer Expertise:

NA

I confirm that I have read this submission and believe that I have an appropriate level of expertise to confirm that it is of an acceptable scientific standard.

# Reviewer report: Reviewer response for version 3

**DOI:** 10.5256/f1000research.193459.r441778

**Published:** 2025-12-31

**Authors:** Nawaz Haider Bashir

**Affiliations:** 1 Qujing Normal University, Qujing, Yunnan, China

The authors have incorporated the necessary changes in the revised version.

Are the datasets clearly presented in a usable and accessible format, and the assembly and annotation available in an appropriate subject-specific repository?

Partly

Are sufficient details of the sequencing and extraction, software used, and materials provided to allow replication by others?

Partly

Are the rationale for sequencing the genome and the species significance clearly described?

Yes

Are the protocols appropriate and is the work technically sound?

Yes

Reviewer Expertise:

Entomologist, Evolutionary Biology

I confirm that I have read this submission and believe that I have an appropriate level of expertise to confirm that it is of an acceptable scientific standard.

# Reviewer report: Reviewer response for version 3

**DOI:** 10.5256/f1000research.193459.r441779

**Published:** 2025-12-16

**Authors:** Hoang Dang Khoa Do

**Affiliations:** 1 Nguyen Tat Thanh University, Ho Chi Minh City, Ho Chi Minh, Vietnam

Dear Authors,

 Thank you very much for revising the manuscript.

 However, many issues were not addressed, and the text was the same as previous version without clear explanation.

 Therefore, I am not confident for further reviewing this manuscript.

 Best regards.

Are the datasets clearly presented in a usable and accessible format, and the assembly and annotation available in an appropriate subject-specific repository?

Partly

Are sufficient details of the sequencing and extraction, software used, and materials provided to allow replication by others?

Yes

Are the rationale for sequencing the genome and the species significance clearly described?

Partly

Are the protocols appropriate and is the work technically sound?

Yes

Reviewer Expertise:

Genomics, Chloroplast genome evolution, Phylogeny

I confirm that I have read this submission and believe that I have an appropriate level of expertise to state that I do not consider it to be of an acceptable scientific standard, for reasons outlined above.

# Reviewer report: Reviewer response for version 2

**DOI:** 10.5256/f1000research.166509.r424130

**Published:** 2025-11-20

**Authors:** Zinian WU

**Affiliations:** 1 Chinese Academy of Agricultural Sciences, Beijing, Beijing, China

1. The description of sequencing in the Methods section is excessively detailed, and most content pertains to routine operations performed by the sequencing company. It is recommended to condense this part: briefly state the sequencing method, data volume, and other core information in one sentence, and focus on the analysis and discussion of the results.

 2. The chloroplast genome typically has a quadripartite structure, consisting of four regions (LSC, SSC, IRa, and IRb), where IRa and IRb are equal in length and inverted in direction. However, the lengths of the two IR regions assembled by the authors are 20,121 bp and 20,115 bp, respectively, which is obviously incorrect. Consequently, the annotation results are inevitably inaccurate, and the authors are advised to further verify and correct them.

 In summary, it is recommended to reject the manuscript and invite resubmission after revisions.

Are the datasets clearly presented in a usable and accessible format, and the assembly and annotation available in an appropriate subject-specific repository?

Partly

Are sufficient details of the sequencing and extraction, software used, and materials provided to allow replication by others?

Yes

Are the rationale for sequencing the genome and the species significance clearly described?

No

Are the protocols appropriate and is the work technically sound?

Yes

Reviewer Expertise:

genome

I confirm that I have read this submission and believe that I have an appropriate level of expertise to state that I do not consider it to be of an acceptable scientific standard, for reasons outlined above.

# Reviewer report: Reviewer response for version 2

**DOI:** 10.5256/f1000research.166509.r421284

**Published:** 2025-11-12

**Authors:** Nawaz Haider Bashir

**Affiliations:** 1 Qujing Normal University, Qujing, Yunnan, China

This Genome Note describes the re-sequencing and assembly of the chloroplast genome of *Cinnamomum burmanni* from Indonesia. The topic has significant relevance and potential value, particularly since it adds geographic representation to existing plastome data. The authors have already revised and improved the manuscript in accordance with the reviewers’ comments. However, the manuscript still requires substantial improvements, such as:

 In the introduction, it is not clear why resequencing the plastome is necessary since there is already a complete *C. burmanni* plastome (China accession).

 Remove repeated sentences in the introduction, like "MinION, one of the ONT devices."

 Differences between Indonesian and Chinese accessions and their biological significance need to be explained.

 Figure 1: ycf1 appears in Figure 1 but is missing in Table 1.

 Results of sequencing can be made clear with total reads, average read length, longest read, percentage of reads used for cp assembly, and chloroplast vs. nuclear read percentages.

Are the datasets clearly presented in a usable and accessible format, and the assembly and annotation available in an appropriate subject-specific repository?

Partly

Are sufficient details of the sequencing and extraction, software used, and materials provided to allow replication by others?

Partly

Are the rationale for sequencing the genome and the species significance clearly described?

Yes

Are the protocols appropriate and is the work technically sound?

Yes

Reviewer Expertise:

Entomologist, Evolutionary Biology

I confirm that I have read this submission and believe that I have an appropriate level of expertise to confirm that it is of an acceptable scientific standard, however I have significant reservations, as outlined above.

# Reviewer report: Reviewer response for version 2

**DOI:** 10.5256/f1000research.166509.r417958

**Published:** 2025-10-11

**Authors:** Minh Trong Quang

**Affiliations:** 1 University of Medicine and Pharmacy, Ho Chi Minh City, Vietnam

Your Genome Note is a useful contribution that documents a MinION-based chloroplast assembly for Indonesian *Cinnamomum burmanni*, but to make it scientifically sound and maximally reusable, please

 (i) strengthen the rationale by stating explicitly what ONT long reads add beyond the existing Chinese plastome (e.g., resolving repeats, verifying IR junctions, detecting structural/indel differences) and include a concise, tabulated comparison (genome size, GC%, LSC/SSC/IR lengths, IR junction coordinates, gene content);

 (ii) deposit the assembled plastome (FASTA) and its annotation (GenBank flatfile and/or GFF3) in a subject-specific repository (GenBank/ENA/DDBJ) and cite the accession(s) in the Data Availability section, alongside a clear list of all deposited file types (FASTQ and, if possible, FAST5/POD5; assembly FASTA; GFF3/GBK; circular genome map) with checksums/DOIs;

 (iii) resolve the IR asymmetry (IRa vs IRb lengths) by validating junctions with read-depth plots and alignment/dot-plots—either justify true asymmetry or correct the assembly—and report the outcome briefly in Results/Discussion; (iv) improve reproducibility by providing key commands/parameters for each step (Guppy model and thresholds; read filtering; Flye options including genome size/plasmid mode; number of polishing iterations and Medaka model; circularization/rotation procedure; GeSeq reference sets and identity thresholds; any manual curation of starts/stops and introns), plus a simple pipeline schematic;

 (v) add minimal HMW DNA QC evidence (method and metrics, image or table) to support the reported read N50 and correct the MinKNOW wording to reflect its role during acquisition rather than post-run visualization;

 (vi) ensure internal consistency between Figure 1 and Table 1 (e.g., presence of ycf1; remove “fragment” labels), standardize numerical formatting (e.g., 152,765 bp), and trim repeated sentences in the Introduction; and

 (vii) enrich the Results with mapping proportion to chloroplast, percent reads retained after filtering, basic assembly stats, and a coverage-depth plot across the plastome. These revisions will clarify the manuscript’s added value, secure data accessibility, and substantially improve technical rigor and reproducibility.

Are the datasets clearly presented in a usable and accessible format, and the assembly and annotation available in an appropriate subject-specific repository?

Partly

Are sufficient details of the sequencing and extraction, software used, and materials provided to allow replication by others?

Partly

Are the rationale for sequencing the genome and the species significance clearly described?

Partly

Are the protocols appropriate and is the work technically sound?

Partly

Reviewer Expertise:

Bioinformatics, Genetics, Biotechnology

I confirm that I have read this submission and believe that I have an appropriate level of expertise to state that I do not consider it to be of an acceptable scientific standard, for reasons outlined above.

# Reviewer report: Reviewer response for version 2

**DOI:** 10.5256/f1000research.166509.r280927

**Published:** 2024-06-11

**Authors:** Hoang Dang Khoa Do

**Affiliations:** 1 Nguyen Tat Thanh University, Ho Chi Minh City, Ho Chi Minh, Vietnam

Dear Authors,

 Thank you very much for your revised manuscript.

 The manuscript was revised based on the reviewer's comments.

 However, some issues have not been resolved.

 1) The d 152.775 bp in the text. unequal length of IR regions was not discussed with the reason.

 2) Comparison between the China and Indonesia *Cinnamomum burmanni* is too simple. Please add more details.

 3) Figure 1 was not revised (please delete "fragment" in the gene names).

 Other comments for minor issues.

 1) ycf1 gene was shown in Figure 1, but it is absent in Table 1.

 2) Please use the format of 152,765 bp and 152,775 bp for 152.765 bp an

 Thank you.

Are the datasets clearly presented in a usable and accessible format, and the assembly and annotation available in an appropriate subject-specific repository?

Partly

Are sufficient details of the sequencing and extraction, software used, and materials provided to allow replication by others?

Yes

Are the rationale for sequencing the genome and the species significance clearly described?

Partly

Are the protocols appropriate and is the work technically sound?

Yes

Reviewer Expertise:

Genomics, Chloroplast genome evolution, Phylogeny

I confirm that I have read this submission and believe that I have an appropriate level of expertise to confirm that it is of an acceptable scientific standard, however I have significant reservations, as outlined above.

# Reviewer report: Reviewer response for version 2

**DOI:** 10.5256/f1000research.166509.r280926

**Published:** 2024-06-11

**Authors:** Jakub Sawicki

**Affiliations:** 1 Department of Botany and Nature Protection, University of Warmia and Mazury in Olsztyn, Olsztyn, Poland

The revised version has improved, but it still requires some corrections.

 Introduction: The last two sentences should be removed because they repeat previous statements.

 Methods Section:

     1. MinKnow is not intended for post-run visualization.

     2. The authors did not assess HMW DNA fragment distribution, which raises questions about how they determined the N50 value and whether their results accurately reflect this metric.

Are the datasets clearly presented in a usable and accessible format, and the assembly and annotation available in an appropriate subject-specific repository?

Partly

Are sufficient details of the sequencing and extraction, software used, and materials provided to allow replication by others?

Partly

Are the rationale for sequencing the genome and the species significance clearly described?

Partly

Are the protocols appropriate and is the work technically sound?

Partly

Reviewer Expertise:

Chloroplast genomics, nanopore sequencing

I confirm that I have read this submission and believe that I have an appropriate level of expertise to confirm that it is of an acceptable scientific standard, however I have significant reservations, as outlined above.

# Reviewer report: Reviewer response for version 1

**DOI:** 10.5256/f1000research.159786.r262304

**Published:** 2024-04-25

**Authors:** Jakub Sawicki

**Affiliations:** 1 Department of Botany and Nature Protection, University of Warmia and Mazury in Olsztyn, Olsztyn, Poland

This paper presents a clear and well-structured investigation into the chloroplast genome of Indonesian Cinnamomum burmanni. The authors effectively introduce the plant's significance and the rationale for studying its genome, highlighting the knowledge gap regarding this specific Indonesian variety. Authors propose the use of MinION Oxford Nanopore Technologies for sequencing and provide a detailed, step-by-step methodological outline. This includes sample collection, DNA extraction techniques, sequencing hardware, and a comprehensive list of software used for analysis. While a bit more context on chloroplast genome research and the reasoning behind specific methodological choices would be beneficial, the paper overall offers a solid foundation for understanding their research aims and procedures.

 However the reviewed Genome Note has some issues that should be solved before acceptance:

 1. The justification of study is unclear since the reference genome was previously sequenced. Why the ONT sequencing was used and what are possible pros of using this approach

 2. Meanwhile the second genome is reported, the paper lacks of any comparison between Chinese and Indonesian accessions.

 3. The described DNA extraction protocol is not suitable for HMW DNA. There's no size distribution or validation at this point.

 4. The results section lack of any details on sequencing results including total output, percentage of read used for assembly, N50 reads and assembly coverage.

 5. How the authors handle heteroplasmy?

 6. An the least but not last, the SRA accesssion data do not contain fastq/fast5/pod5/ reads.

Are the datasets clearly presented in a usable and accessible format, and the assembly and annotation available in an appropriate subject-specific repository?

Partly

Are sufficient details of the sequencing and extraction, software used, and materials provided to allow replication by others?

Partly

Are the rationale for sequencing the genome and the species significance clearly described?

Partly

Are the protocols appropriate and is the work technically sound?

Partly

Reviewer Expertise:

Chloroplast genomics, nanopore sequencing

I confirm that I have read this submission and believe that I have an appropriate level of expertise to state that I do not consider it to be of an acceptable scientific standard, for reasons outlined above.

# Response

**Published:** 2024-05-13

**Authors:** Richard Salindeho

Dear Editor and Reviewer,

 Thank you for your comment and suggestion.

 We have responded to all reviewer's comment's as you may find in the following link

Response to Reviewer's comments

 Sincerely yours,

 Corresponding author

# Reviewer report: Reviewer response for version 1

**DOI:** 10.5256/f1000research.159786.r255270

**Published:** 2024-03-18

**Authors:** Hoang Dang Khoa Do

**Affiliations:** 1 Nguyen Tat Thanh University, Ho Chi Minh City, Ho Chi Minh, Vietnam

1/ "MinION is a third-generation sequencing technology with nanopore technology (Oxford Nanopore Technologies (ONT), 2017)". MinION is one of the devices of ONT for performing sequencing. Therefore, please revise the sentence.

 2/ Please revise the Figure 1. There are several errors in gene names such as three ndhK, rrn16 fragment, rps12 absence, etc...

 3/ Also, please revise the Figure 1 legend with more detail about Figure 1.

 4/ The protocol for DNA extraction can be found in the kit. Therefore, mentioning it again in the method part is unnecessary. The authors should add more details about the results and comparison between the new and old chloroplast genomes of *Cinnamomum burmanni.*

 5/ Please add the coverage depth of the new chloroplast genome.

 6/ The IR regions do not have the same length. Please add the reason for this uncommon in chloroplast genome.

Are the datasets clearly presented in a usable and accessible format, and the assembly and annotation available in an appropriate subject-specific repository?

Partly

Are sufficient details of the sequencing and extraction, software used, and materials provided to allow replication by others?

Yes

Are the rationale for sequencing the genome and the species significance clearly described?

Partly

Are the protocols appropriate and is the work technically sound?

Yes

Reviewer Expertise:

Genomics, Chloroplast genome evolution, Phylogeny

I confirm that I have read this submission and believe that I have an appropriate level of expertise to confirm that it is of an acceptable scientific standard, however I have significant reservations, as outlined above.

# Response

**Published:** 2024-04-07

**Authors:** Richard Salindeho

Dear Reviewer,

 Thank you for the constructive inputs to our manuscript.

 We have responded to all reviewer's comment's as you may find in the following link

Response to Reviewer's comments

 Please note that the revised Figure 1 is presented in this link

 Best Regards.

 Richard Andreas Salindeho
